# The Patterns of Caregiving Activities for Family Caregivers of Older Adults: A Latent Class Analysis

**DOI:** 10.1093/geroni/igaa057.1141

**Published:** 2020-12-16

**Authors:** Jing Huang, Pui Hing Chau, Edmond Pui Hang Choi, Bei Wu, Vivian Lou

**Affiliations:** 1 The University of Hong Kong, Hong Kong, China; 2 New York University, New York, New York, United States

## Abstract

The purposes of this study were to identify the patterns of caregiving activities among family caregivers in Hong Kong and to examine their associations with characteristic factors and caregiver burden. The data was from the cross-sectional survey on the profiles of family caregivers of older adults in Hong Kong. 932 family caregivers were classified into different classes by using the Latent class analysis (LCA) according to their engagements in the 17 daily caregiving activities: 6 activities of daily living (ADLs), 8 instrumental activities of daily living activities (IADLs), emotional support, decision-making, and financial support. Five classes were revealed and labeled “Total All-round Caregiving” (Class I: 19.5%), “Partial All-round Caregiving” (Class II: 8.2%), “ADLs Free Caregiving” (Class III: 23.8%), “ADLs & Partial IADLs Free Caregiving” (Class IV: 32.5%), “Financial Caregiving” (Class V: 16.0%), respectively. Results from multinomial logistic regression found that the following factors were associated with the class membership: care recipients’ age, medical diagnoses, and caregivers’ gender, job status, marital status, self-rated economic status, living with care recipients, and caring for ≥40 hours per week. Findings from multiple linear regression showed caregivers with different patterns of caregiving activities reported different levels of caregiver burden. Caregivers in Class I have been found with the highest caregiver burden. This is the first study that has applied LCA to capture the patterns of caregiving activities among family caregivers. Identification of caregiving activity patterns and examination of their characteristics and caregiver burden can help healthcare providers to shift to prioritized and targeted caregiver support.

